# Determining the Need for Palliative Care Patients with Multiple Sclerosis—A Cross-Sectional Study

**DOI:** 10.3390/healthcare12202024

**Published:** 2024-10-11

**Authors:** Branimirka Aranđelović, Svetlana Simić, Dragana Simin, Milena Mikić, Vladimir Dolinaj, Slobodanka Bogdanović Vasić, Dragana Milutinović

**Affiliations:** 1 Department of Nursing, Faculty of Medicine, University of Novi Sad, 21000 Novi Sad, Serbia; milena.mikic@mf.uns.ac.rs (M.M.); vladimir.dolinaj@mf.uns.ac.rs (V.D.); dragana.milutinovic@mf.uns.ac.rs (D.M.); 2 Department of Neurology, Faculty of Medicine, University of Novi Sad, 21102 Novi Sad, Serbia; svetlana.simic@mf.uns.ac.rs; 3 Department of Medical and Business-Technological Studies, Academy of Vocational Studies, 15000 Šabac, Serbia; s.bogdanovicvasic@gmail.com

**Keywords:** palliative care, chronic disease, multiple sclerosis, needs assessment, functional dependence, end-of-life care

## Abstract

Background/Objectives: Multiple sclerosis is characterised by the manifestation of heterogeneous symptoms that affect daily functioning. Patients face physical impairment, psychological problems and socioeconomic changes. Depending on the form of the disease, they may have different needs, which are often unsatisfied and could be overcome by including palliative care in the treatment. Despite the above, not enough is known about this population’s needs for palliative care. This cross-sectional study aimed to identify the need for palliative care and assess the functional dependence level in daily living activities in patients with multiple sclerosis. Methods: The sample consisted of 120 patients from the Neurology Clinic of the University Clinical Center of Vojvodina, Serbia. The following research instruments were used: a General questionnaire, Sheffield Profile for Assessment and Referral for Care, Multiple Sclerosis Impact Scale and Barthel Index. Results: The need for palliative care was expressed by 36.7% of patients, who expressed the most concern for the physical symptoms, independence and activity domains. Also, it was determined that the disease prevents patients from doing demanding physical tasks and that they are worried about multiple sclerosis. Almost a quarter of patients have had complete/severe dependence on other persons in performing daily activities. Conclusions: The most frequently reported physical symptoms by patients with MS were weakness, bladder problems, fatigue and different levels of dependence, while concerns related to sexual health, low mood and anxiety emerged as key challenges in the psychological domain. These findings underscore the importance of conducting needs assessments to guide the development of an appropriate palliative care model for MS patients.

## 1. Introduction

Multiple sclerosis (MS) is a chronic, inflammatory, neurodegenerative and autoimmune disease of the central nervous system [1,2,3]. It is characterised by physical, neurological, and often cognitive outbursts and functional limitations [4,5]. Signs and symptoms of the disease range from mild to severe [1], depending on the location of the demyelinating lesions [3]. Clinical manifestations usually include motor symptoms, sensory disturbances, vision problems, urinary and intestinal system dysfunction [2,4,6] and highly prevalent fatigue [1,3]. The progression of MS is variable [1], and it mainly depends on the patient’s disease type. There are four types of MS: relapsing-remitting (RRMS), primary-progressive (PPMS), secondary-progressive (SPMS) and progressive-relapsing (PRMS) [4]. According to data from 2023, approximately 2,900,000 people will be affected by MS worldwide [7]. It is more common in females than males (3:1) [3]. In most cases, MS begins between the ages of 20 and 40 [1,2,3,4]. The importance of MS is indicated by the fact that the onset of the disease is related to young people and that it is the most common cause of non-traumatic disability in this population [1,3,4].

The progression of the disease leads the patients to a reduced ability to perform daily activities and difficult participation in numerous domains of life [8,9], including family, social and economic functioning [5]. All this leads to a significant deterioration of the patient’s quality of life [1,8,9].

With the progression of the disease, treating complex symptoms and needs of patients requires a holistic approach involving a multidisciplinary healthcare team [3,6,10,11] and palliative care provision [3,11,12]. However, despite the comparable severity of symptoms between individuals with advanced multiple sclerosis (MS) and those with advanced cancer, palliative care services for MS patients remain inadequate in many countries, facing numerous challenges. As a result, only a small proportion of MS patients have access to these essential services [11]. To provide palliative care services adequately, healthcare professionals must identify and define the needs of MS patients [10]. Therefore, this study aimed to identify the need for palliative care and assess the functional dependence level in daily living activities in patients with MS.

## 2. Materials and Methods

### 2.1. Study Setting and Sampling

The study was designed as a cross-sectional study by surveying patients of the Clinic for Neurology, University Clinical Center of Vojvodina, Serbia, who had been hospitalised or undergoing a follow-up examination for one year. The study sample included a total of 120 patients with MS. The criteria for inclusion in the study were patients aged ≥ 18, both genders, with a diagnosis of multiple sclerosis and signed voluntary informed consent of the subjects. Criteria for non-inclusion in the research were patients under 18 years of age, patients with associated malignant diseases, patients who cannot communicate effectively, and the patient’s non-consent to the research. The study adhered to Strengthening the Reporting of Observational Studies in Epidemiology (STROBE) guidelines.

### 2.2. Instruments

Sociodemographic data on the patients were collected using a general questionnaire designed for this study, which consists of two parts. The first part refers to the basic patient’s demographic characteristics and the second part to the clinical characteristics of MS.

The Sheffield Profile for Assessment and Referral for Care (SPARC-45 v1.1) questionnaire, designed in 2008 by the Academic Unit of Supportive Care, University of Sheffield, was used to assess patients’ palliative care needs. SPARC is used to evaluate needs and holistically identify patients needing palliative care. The questionnaire can be used independently of the disease diagnosis [13]. The SPARC consists of 45 items covering eight domains of potential needs, including communication and information issues, physical symptoms, psychological issues, religious and spiritual issues, independence and activity, family and social issues, treatment issues and personal issues [13,14]. The items from two domains were answered with Yes/No, and on the other domains, they were divided into four categories from 0 to 3 (not at all to very much) [14]. In our study, the Cronbach alpha coefficient for the SPARC questionnaire in patients with multiple sclerosis was 0.93. Permission was obtained from the author to translate the SPARC questionnaire into Serbian and use it in this study.

The Multiple Sclerosis Impact Scale—MSIS-29 (version 1) was designed in 2001 [15]. The scale measures multiple sclerosis’s physical and psychological impact on daily life from the patient’s point of view in the previous 14 days. It contains 29 items, with answers provided on a five-point Likert scale from 0 to 5 (from not at all to extreme). A higher score indicates a higher degree of disability [16]. Permission to use the MSIS-29 scale in Serbian was obtained from the author of the scale.

Barthel Index—BI, version 1.0 [17] is used to assess the functional status (activities of daily living (ADL) and mobility). The assessment of (non)dependence, i.e., patient dysfunction, is assessed through 10 items: feeding, dressing and undressing, maintaining personal hygiene, bathing, using the toilet, control of bowel movements, control of bladder movements, mobility, movement from wheelchair to bed and back and, going up and down the stairs. The sum of the points of all items represents the total score, which ranges from 0 (completely dependent) to 100 (completely independent). A higher score indicates a greater degree of independence. Interpretation of the results: 0 to 20 complete dependence, 21 to 60 severe dependence, 61 to 90 moderate, 91 to 99 low dependence and 100 complete independence [18]. Permission to use and translate Barthel’s index into Serbian was obtained from the Mapi Research Trust, France.

### 2.3. Data Analysis

Statistical data analysis was performed using the statistical programme IBM SPSS Statistics 21.0. Data processing included methods of descriptive and inferential statistics. Numerical characteristics are presented through mean values (arithmetic mean), variability (standard deviation) measures and attributive characteristics using frequencies and percentages. Student’s *t*-test was applied to compare the average values for the numerical features of the two groups. One-way analysis of variance (ANOVA) was applied to test differences in the average values of three or more data groups, and further mutual comparison was performed using the Tukey test. Pearson’s correlation coefficient (r) and Spearman’s correlation coefficient (ρ) were used to test the association of variables. Cronbach’s alpha coefficient was used to check the reliability of The Sheffield Profile for Assessment and Referral for Care (SPARC-45 v1.1) questionnaire. All tests are two-sided with a significance level of *p* < 0.05.

### 2.4. Ethical Considerations

This study was conducted following the Helsinki Declaration on ethical principles involving human subjects and was approved by the Ethics Committee of the University Clinical Center of Vojvodina (Number 00-20/382). Before conducting the study, the patients received written information about the study’s aims and the procedure for collecting and storing data. For voluntary participation in the study, patients signed the Informed Consent form.

## 3. Results

### 3.1. Sociodemographic Characteristics of Patients

The study included a total of 120 patients with MS aged 30 and over, of which almost two-thirds were female. The sociodemographic and clinical characteristics of the patients are presented in Table 1.

### 3.2. Need for Palliative Care among Patients with MS

Table 2 shows patients’ most common complaints from the SPARC questionnaire. Physical symptoms that affected MS patients quite a lot are feeling weak (50.0%) and bladder problems (48.3%), psychological issues, the effect of the disease on sex life (36.7%) and the feeling of a low mood (26.7%). As for the independence and activity domain, it is the inability to perform usual household chores (40.9%).

In the domain of communication and information issues, patients stated that they were most able to talk with the family (92.5%), the doctor (90.0%), hospital nurses (66.7%), community nurses (50.0%) and other people (64.2). In the domain of personal issues, more than half of the patients reported that they would like more information about their condition, care and treatment (55.0%, 55.8% and 56.7%), a third about other types of support (35.8%) and more than a quarter about financial issues (27.5%). The need for help with personal affairs was expressed by (70%) of patients, and the need to talk to another professional about their condition/treatment (60.0%).

Based on answers to questions from all domains of the SPARC questionnaire, the need for palliative care was reported by 36.7% of patients with MS.

Table 3 presents the descriptive parameters of the Multiple Sclerosis Impact Scale (MSIS-29, version 1) and its subscales (Physical Impact and Psychological Impact), with the scores transformed to a 0–100 scale.

By analysing individual items from the physical impact subscale of the MSIS-29 scale, it was determined that MS most limited the patient’s ability to do demanding physical tasks and carry things. Furthermore, they were disturbed by the need to go to the toilet urgently, and from the psychological subscale, they expressed worries related to their MS. The most prevalent complaints reported by patients related to the physical and psychological impact of MS, which is specific to the disease, are shown in the Table S1 attached in the Supplementary Materials.

### 3.3. Differences Concerning the Type of MS and Comorbidities

By analysing the MSIS-29 scale concerning the type of MS, we obtain the data shown in Table 4. Further analysis (Tukey test) revealed a significantly higher MSIS-29 total score in patients with primarily progressive compared to the relapsing-remitting type of MS (*p* < 0.001), as well as in patients with secondary progressive compared to relapsing-remitting disease (*p* = 0.001). Significantly higher average scores on the physical impact of MS were found in patients with primarily progressive compared to relapsing-remitting disease (*p* < 0.001), as well as in patients with secondary progressive disease compared to relapsing-remitting (*p* = 0.001). The average scores of the psychological impact of MS were significantly higher in patients with primarily progressive compared to patients with relapsing-remitting type (*p* = 0.006). At the same time, no significant difference was found between patients with secondary progressive and relapsing-remitting disease (*p* = 0.107). Also, no significant difference was found in the scores of the MSIS-29 total score (*p* = 0.998), the physical impact (*p* = 0.992) and the psychological impact of MS (*p* = 0.995) between patients with primary progressive and secondary progressive disease.

Table 5 shows the impact of comorbidities on MS patients.

### 3.4. Level of Functional Dependence in Daily Life Activities

Table 6 shows the distribution of functional dependence of MS patients obtained using the Barthel index.

### 3.5. Correlation of the SPARC Questionnaire and the MSIS-29 v1 Scale

Table 7 shows the correlation between the MSIS-29 v1 total score, the physical and psychological impact of MS, the SPARC total score and the score on its domains. A significant and positive correlation was determined for all the variables.

### 3.6. Correlation of the Barthel Index and the Independence and Activity Domains of the SPARC Questionnaire

To examine the correlation between the Barthel Index and the Independence and Activity domain of the SPARC questionnaire (items 34, 35, and 36) in patients with MS, Pearson’s correlation coefficient (r) and Spearman’s correlation coefficient (ρ) were applied. Both coefficients revealed a significant negative correlation (r = −0.836, *p* < 0.001; ρ = −0.821, *p* < 0.001).

### 3.7. Correlation of the MSIS-29 v1 Scale and Barthel’s Index (Level of Functionality)

The level of functionality is shown in the form of three categories: complete/severe dependence, moderate dependence and low dependence/complete independence. The average score of the MSIS-29 total score was significantly higher, i.e., the degree of disability is higher in patients with a higher degree of functional dependence (F = 69.723, *p* < 0.001), as well as in the subscale MSIS 29—physical impact (F = 88.260, *p* < 0.001) and the subscale MSIS 29—psychological impact (F = 10.713, *p* < 0.001). Analysis of the Multiple Sclerosis Impact Scale (MSIS-29 v1), total score and physical and psychological impact concerning the level of functionality is shown in Table 8.

## 4. Discussion

This study highlights the challenges faced by patients with MS, emphasising their need for palliative care and the extent of their functional dependence on daily activities. Our findings reveal that over one-third of patients expressed a need for palliative care, with the most pressing issues falling within the domain of physical symptoms, particularly weakness, bladder problems and fatigue. This was followed by the Independence and Activity domains, where patients with MS struggled with routine household tasks, and the Psychological Problems domain, where issues such as the impact of the disease on sexual life and feelings of low mood were reported. In contrast, they experienced significantly fewer difficulties in the areas of Family and Social Issues, Religious and Spiritual Concerns and Treatment-Related Issues.

Analysing the items of the physical impact of MS from the MSIS-29 scale that is specific to the disease, it was determined that MS most limited the ability of patients to do demanding physical tasks, to carry things and they were disturbed by the urgent need to go to the toilet. Patients often complained of paraesthesia, but the scale does not include that question, so we do not have precise data on the frequency of that symptom. Worries about MS, the impression that they do not feel well, depression, feelings of worry and irritability were most often reported by patients in the psychological impact subscale.

Our results align with another study regarding fatigue and weakness in patients. Other studies also highly represent physical or mental fatigue [1,19,20,21,22] and weakness [19]. Fatigue impacts many domains of life, impairing the quality of life [19,20]. Depression was also reported in a high percentage (81.9%) [19]. Depression can occur at any stage of MS, in all age categories, with the highest frequency in the secondary progressive type of the disease. The prevalence of depression ranges from 16% to 41%. Study data show that it is not correlated with the duration of the disease nor with functional impairment. Besides depression, a high percentage of anxiety occurs (34%), especially pronounced at the beginning of the disease [23], which is confirmed by other research [12].

Other studies report numbness, spasticity, difficulties with vision, balance, motor skills, bladder, bowel, speech and sexual dysfunction among the complaints faced by MS patients [22]. Most patients have bladder problems due to the disease [24]. The study by Dourou et al. [25] included 130 women with MS aged 35 to 50 years, intending to determine the impact of MS on sexual function and quality of life. The study results showed sexual dysfunction in patients and a significant correlation between sexual dysfunction and low quality of sexual life, as well as increased fatigue with reduced sexual satisfaction. Also, in this study, it was determined that demographic factors had an impact on sexual functioning and that fatigue was a significant predictor of sexual dysfunction.

Many studies show improvement and relief of certain symptoms in these patients after including palliative care interventions [10,21].

Social support is an important aspect that directly impacts preserving people’s health and quality of life. In difficult life situations, there is an increased need for social support. The previous study results show the psychophysical benefits of the patients who received various forms of social support. Also, it was discovered that reduced mobility in patients with MS contributes to a lower amount of social support provided to these patients, which makes them feel useless and helpless, with the presence of a lower degree of self-esteem [22]. A family’s life changes from its roots when one of its members is diagnosed with MS. The basic challenges of family life that arise in that case include changing roles, parenting issues, patterns of family communication, joint grieving processes, conflicting styles of coping with the disease and adaptation and reorganisation [26]. For critically ill patients, important aspects are reorganising everyday life, restructuring social roles in family and professional life and preserving personal identity [12]. Also, patients with MS have an increased need for emotional support [10], given that, as with many chronic diseases, emotions in the form of guilt, sadness, concern and frustration occur [27].

Every patient who has MS should be informed about the diagnosis, nature of the disease, prognosis and treatment options. In one study, 70% of patients with MS and 83% of their family members or friends agreed that the patient should be informed about the diagnosis of the disease [28]. The study’s results by Galushko et al. [29] show dissatisfaction with the quality of interaction between the doctor and the patient. Patients with MS would like more time to talk with doctors, more adequate communication of bad news, to be observed individually with more empathy and compassion and to be listened to more. Also, they would like assistance from their doctors regarding the interpretation of the information they have come up with, and they would like more information about the disease, its progression, treatment options, and different available services. The authors noted that healthcare professionals involved in patients with MS care must provide support and information to them with empathy so that they can more easily face the life changes caused by the disease, as well as its consequences. In our study, most patients could talk to their family, their chosen doctor and nurses. Also, more than half of the respondents wanted more information about their condition, care and treatment.

Regarding free time, in one study, patients with MS reported no alternative to the activities they enjoyed before they became sick, such as sports or travel [29]. The results of our study show that the disease limits social activities and leisure activities that patients perform at home.

Support from family and friends, health services, managing daily life and maintaining biographical continuity are identified as unmet needs from the point of view of health professionals [30]. A study with similar results showed unmet needs from the patient’s point of view. They point to unsatisfied needs from many domains of life (support of family and friends, daily activities, free time, employment) and healthcare services (difficulties accessing health services, long distances from specialists, long waiting times, transportation services). In another survey, they expressed dissatisfaction with diagnosis management, availability of care, quality of care, unnecessary complications, insufficient psychological support, information deficit and inadequate coordination between services [31]. In order to meet the needs of patients, it is concluded that it is necessary to improve health services and better coordination with palliative care services [29].

Forbes et al. [31], by analysing the results from 23 studies, the needs of patients with MS were categorised as psychological (control of symptoms, adaptation to the disease), social (employment, finances, stigma), physical (control of symptoms, daily activities) and knowledge about the disease and availability of a health professional. They grouped the identified needs of MS patients into seven categories: medical treatment (symptom control, treatment), socio-ecological support and adaptation (environmental adaptation, finances, employment, transportation), provision of improved care (availability and continuity of health and social care provision), provision of information (more information about disease treatments and services), rehabilitation therapies (greater access to physiotherapy, provision of rehabilitation facilities), non-professional care (support for personal, child and home care) and psychological support (access to help for psychological issues). Priorities of needs depended on the level of disease impact.

Dadsetan et al. [32] compared palliative care needs from the perspective of patients with MS and nurses employed in neurological departments. Participants from both groups emphasised the physical domain of palliative care, followed by social, spiritual, psychological and economic. However, patients prioritised their needs from all domains more than nurses. Their results suggest that patients with MS need palliative care from all domains and that they should be given access to palliative care that meets their individual needs.

Multiple sclerosis leads to a decline in functional status (motor, sensory, cognitive, behavioural and communication), which can occur due to physical disability, fatigue, pain, incontinence and cognitive and psychological difficulties. The mentioned manifestations result in limitations in daily functioning [6] and the loss of the ability to live independently, which results in increased dependence on other people and impairment of the quality of life. In most cases, symptoms that appear in the initial phase of MS do not interfere with activities of daily living (ADL). As the disease progresses, the symptoms become more numerous and pronounced, negatively impacting ADL, as shown by many studies [8].

In our study, functional independence was obtained using the Barthel index, and almost a quarter of patients highly depended on others in their daily activities. The independence and activity domain also obtained this study’s functional ability data in the SPARC questionnaire. The results are consistent with the results obtained in the Bartel index.

Using the Bartel index, Basak et al. [8] found that 79.1% of the patients had low dependence on ADL, and 1.5% had complete dependence. A ten-year study in Sweden, which included 264 subjects with mild, moderate and severe MS, shows that patients with moderate MS significantly increased dependence in most domains from ADL and instrumental ADL. Patients with mild MS increased dependence on instrumental ADL, while patients with severe increased dependence on ADL. In addition, most patients with severe MS show limited participation in all social and life activities from the very beginning of the disease [9]. The study’s results by Galushko et al. [29] showed that the most unmet needs were related to household maintenance and adapting living conditions to their physical needs.

As it is known, patients with MS are a heterogeneous population, where the progression of the disease and symptoms differ from each other, and therefore the needs of the patients are different. Neurological treatment is insufficient to meet all needs; therefore, including palliative care, especially in treating seriously ill patients, can be valuable [10]. Studies have shown that patients with severe MS have many different unsatisfied needs, which affect their physical, psychological [29] and social domains. In addition, they face isolation, loss of independence, changes in self-confidence levels, social roles, lack of information and difficulties in using health services [10]. Needs assessment is essential for designing the concept of palliative and neurological care in patients with MS, and multidisciplinary services should be accessible to patients, including an individual approach and be adapted to the needs of patients [29].

### Strengths and Limitations

Although our study is one of the few that assess the palliative needs of MS patients and the study results contribute to the science of palliative care, there are several limitations. The sample consisted of MS patients treated only at one neurological clinic in the Republic of Serbia, so the study results cannot be generalised to the entire population of patients. In addition, the needs of the patients may differ concerning the type and stage of the disease, and it would be necessary to monitor the patients for a longer period, which was not possible in this study, given that the study was designed as a cross-sectional study.

Currently, there is not enough research on this topic, and it is necessary to conduct more studies on a larger sample and a more diverse population in order to assess the needs and priorities of MS patients depending on the type and stage of the disease, identify the best time to integrate palliative care into treatment, and in accordance thereby providing appropriate palliative services. In addition, it is necessary to design an instrument to assess palliative needs specific to MS.

## 5. Conclusions

Palliative care is vital in better symptom management and enhancing patients’ and their families’ quality of life. This study offers valuable insights into the unmet needs of patients with MS, highlighting the potential benefits of integrating palliative care into their treatment. The most frequently reported physical symptoms were weakness, bladder problems and fatigue, while concerns related to sexual health, low mood and anxiety emerged as key challenges in the psychological domain. Additionally, half of the MS patients exhibited moderate dependence, with a quarter experiencing severe or total dependence. These findings underscore the importance of conducting needs assessments to guide the development of an appropriate palliative care model for MS patients.

## Figures and Tables

**Table 1:** Sociodemographic and clinical characteristics of patients with MS.

Variable	n	%
Gender		
Male	48	40.0
Female	72	60.0
Age categories		
30–45	73	60.8
46–65	43	35.8
>65	4	3.3
Average age ($\bar{x}$ ± SD) 43.08 ± 11.84		
Marital status		
Marital/common-law union	59	49.2
They are not married	61	50.8
Live with		
Spouse and/or children	62	51.7
Alone	10	8.3
Caregiver/Parent/Other	48	40.0
Level of education		
Elementary school and below	8	6.7
High school	76	63.3
College/university/postgraduate studies	36	30.0
Work status		
Employed	47	39.2
Unemployed	40	33.3
Pensioner	33	27.5
Patient status		
Hospitalised	108	90.0
Outpatient	12	10.0
Presence of MS in the family		
No	111	92.5
Yes	9	7.5
Types of multiple sclerosis		
Relapsing-remitting MS	92	77.0
Primary-progressive MS	20	17.0
Secondary-progressive MS	7	6.0
Comorbidities		
Arterial hypertension	11	9.2
Hypothyroidism	6	5.0
Epilepsy	5	4.2
Depression	5	4.2

**Table 2:** Distribution of patients with MS on SPARC domains to the most prevalent complaints.

SPARC	Multiple Sclerosis					
	A Little Bit		Quite a Bit		Very Much	
Domains/Items	n	%	n	%	n	%
Physical symptoms						
Pain	52	43.3	11	9.2	5	4.2
Headache	45	37.5	12	10.0	2	1.7
Bowel problems	50	41.7	21	17.5	6	5.0
Bladder problems	30	25.0	39	32.5	19	15.8
Feeling weak	51	42.5	57	47.5	3	2.5
Feeling tired	52	43.3	53	44.2	4	3.3
Concern about changes in appearance	70	58.3	14	11.7	3	2.5
A feeling of restlessness and agitation	49	40.8	26	21.7	5	4.2
Psychological issues						
Feeling anxious	50	41.7	20	16.7	6	5.0
Feeling in a low mood	48	40.0	26	21.7	6	5.0
Feeling confused	46	38.3	16	13.3	4	3.3
Effect on sex life	56	46.7	38	31.7	6	5.0
Religious and spiritual issues						
Thoughts about death and dying	40	33.3	9	7.5	0	0
Independence and activity						
Loss of independence	47	39.2	26	21.7	10	8.3
Inability to perform usual household chores	42	35.0	29	24.2	20	16.7
Family and social issues						
Concern about the consequences of your illness on family and others	75	62.5	28	23.3	2	1.7
Treatment issues						
Concerns about the long-term consequences of therapy	53	44.2	6	5.0	0	0

**Table 3:** Mean and measures of variability of the total of the MSIS-29 v1 and subscales scores.

MSIS-29 v1/Domains (Number Items)	Mean	SD	Med	Min	Max
Physical impact of MS (1–20)	39.52	22.34	38.75	0	87.50
Psychological impact of MS (21–29)	26.13	19.42	19.44	0	86.11
Total	35.37	19.07	34.05	0	83.62

**Table 4:** The total score on the MSIS-29 questionnaire and the scores on the physical and psychological impact of MS: differences concerning type of MS.

A type of Multiple Sclerosis	N	MSIS-29 v1					
		Total Score Mean ± SD	F; *p*	Physical Impact Mean ± SD	F; *p*	Psychic Impact Mean ± SD	F; *p*
Relapsing-remitting	92	29.65 ± 16.51	F = 24.834 *p* < 0.001	32.73 ± 19.52	F = 25.814 *p* < 0.001	22.80 ± 17.54	F = 6.354 *p* = 0.002
Primary progressive	21	54.27 ± 14.79		62.08 ± 16.52		36.90 ± 21.72	
Secondary progressive	7	53.82 ± 14.13		61.07 ± 13.32		37.70 ± 22.56	
Total	120	35.37 ± 19.07		39.52 ± 22.34		26.13 ± 19.42	

**Table 5:** The total score on the MSIS-29 questionnaire and the scores on the physical and psychological impact of MS: differences concerning comorbidity.

Comorbidity	N	MSIS-29 v1					
		Total Score Mean ± SD	t; *p*	Physical Impact Mean ± SD	t; *p*	Psychic Impact Mean ± SD	t; *p*
Without comorbidity	91	33.69 ± 19.01	t = 1.723 *p* = 0.088	38.80 ± 22.94	t = 0.620 *p* = 0.536	22.31 ± 15.74	t = 4.058 *p* < 0.001
Comorbidity	29	40.64 ± 18.59		41.77 ± 20.56		38.12 ± 24.71	
Total	120	35.37 ± 19.07		39.52 ± 22.34		26.13 ± 19.42	

**Table 6:** Distribution of patients with MS to the level of functional dependence.

Barthel Index	Multiple Sclerosis	
	n	%
Total dependence	9	7.5
Severe dependence	18	15.0
Moderate dependence	51	42.5
Little dependence	19	15.8
Complete independence	23	19.2
Total	120	100

**Table 7:** Correlation between the MSIS-29 v1 total score, the physical and psychological impact of MS, the SPARC total score and the score on its domains.

SPARC	MSIS-29 v1		
	Total Score	Physical Impact	Psychological Impact
Physical symptoms	0.715 ***	0.602 ***	0.724 ***
Psychological issues	0.582 ***	0.367 ***	0.905 ***
Religious and spiritual issues	0.396 ***	0.216 *	0.699 ***
Independence and activity	0.759 ***	0.778 ***	0.412 ***
Family and social issues	0.459 ***	0.282 **	0.732 ***
Treatment issues	0.352 ***	0.178 ^ns^	0.657 ***

^ns^ no significant difference, * *p* < 0.05, ** *p* < 0.01, *** *p* < 0.001.

**Table 8:** The total score on the MSIS-29 questionnaire and the scores on the physical and psychological impact of MS: differences concerning the level of functionality.

Level of Functionality	N	MSIS-29					
		Total Score Mean ± SD	F; *p*	Physical Impact Mean ± SD	F; *p*	Psychological Impact Mean ± SD	F; *p*
Total/severe dependence	27	58.24 ± 12.78	F = 69.723 *p* < 0.001	66.34 ± 12.93	F = 88.260 *p* < 0.001	40.23 ± 23.03	F = 10.713 *p* < 0.001
Moderate dependence	51	35.58 ± 12.42		41.45 ± 13.98		22.55 ± 16.10	
Littledependence/complete independence	42	20.40 ± 13.78		19.94 ± 15.27		21.43 ± 16.52	
Total	120	35.37 ± 19.07		39.52 ± 22.34		26.13 ± 19.42	

## Data Availability

All data relevant to the study are included in the article.

## Supplementary Materials

The following supporting information can be downloaded at: https://www.mdpi.com/article/10.3390/healthcare12202024/s1, Table S1: Mean and measures of variability of the most prevalent items of the MSIS-29 v1 scale.
