# Supplementary material for: Determining the Need for Palliative Care Patients with Multiple Sclerosis—A Cross-Sectional Study
Source: Healthcare (Basel). 2024 Oct 11;12(20):2024. doi: 10.3390/healthcare12202024 (PMC11507286; doi:10.3390/healthcare12202024)
Supplement: Supplementary file 1 [file healthcare-12-02024-s001.zip › healthcare-3207105-supplementary.pdf]

SUPPLEMENTARY MATERIALS

Table S1. Mean and measures of variability of the most prevalent items of the MSIS-29 v1 scale

**MSIS-29 v1**<br>**Domains**/Items	**AS**	**SD**	**Med**	**Min**	**Max**
**Physical impact of MS**					
Inability to do demanding physical tasks	3.61	1.16	4.00	1	5
Inability to carry things	3.21	1.30	3.50	1	5
Problems with balance	2.66	1.21	3.00	1	5
Difficulty moving about indoors	2.72	1.30	3.00	1	5
Muscle stiffness	2.58	1.13	3.00	1	5
Heavy arms and/or legs	2.57	1.11	3.00	1	5
Body not doing what they want it to do	2.66	1.06	3.00	1	5
Having to depend on others to do things for them	2.62	1.28	2.00	1	5
Limitations in their social and leisure activities at home	2.50	1.08	2.00	1	5
Being stuck at home more than they would like to be	2.60	1.18	2.00	1	5
Problems using transport	2.78	1.42	3.00	1	5
Taking longer to do things	2.58	1.13	2.00	1	5
Difficulty doing things spontaneously	2.50	1.13	2.00	1	5
Needing to go to the toilet urgently	3.14	1.36	3.00	1	5
**Psychological impact of MS**					
Feeling unwell	2.30	0.85	2.00	1	4
Worries related to their MS	2.41	0.97	2.00	1	5
Feeling anxious or tense	2.24	1.14	2.00	1	5
Feeling irritable, impatient, or short-tempered	2.24	1.16	2.00	1	5
Depressive	2.29	1.32	2.00	1	5
